# Morphology and the central nervous system of *Eratigena atrica* affected by a complex anomaly in the anterior part of the prosoma

**DOI:** 10.1007/s10158-017-0204-0

**Published:** 2017-10-17

**Authors:** Teresa Napiórkowska, Julita Templin, Katarzyna Wołczuk

**Affiliations:** 1 Department of Invertebrate Zoology, Faculty of Biology and Environmental Protection, Nicolaus Copernicus University Toruń, Lwowska 1, 87-100 Toruń, Poland; 2 Department of Vertebrate Zoology, Faculty of Biology and Environmental Protection, Nicolaus Copernicus University Toruń, Lwowska 1, 87-100 Toruń, Poland

**Keywords:** *Eratigena atrica*, Spider, Teratology, Temperature fluctuations, Polymely, Heterosymely

## Abstract

Spider embryogenesis is affected by a range of environmental factors. Any sudden, drastic change in the environment may impair spider development, leading to various body deformities. In the present study, we analyze changes in the morphology and structure of the central nervous system of an *Eratigena atrica* larva, obtained in a teratological experiment in which embryos were exposed to alternating temperatures of 14 and 32 °C for the first 10 days. The studied larva had three pedipalps on the right side of the prosoma (polymely), two of which were fused along their entire length (total heterosymely). In addition, there was a short, club-shaped stump between the pedipalps. Histological analysis confirmed major changes in the structure of the subesophageal ganglion, i.e., the fusion of all three ganglia of pedipalps.

## Introduction

Scientific literature provides many reports of deformities in invertebrates collected in their natural habitat (Buczek 1992; Spanó et al. 2003; Asiain and Márquez 2009; Eeva and Penttinen 2009; Leśniewska et al. 2009; Kozel and Novak 2013; Miličić et al. 2013). Due to the fact that there are many factors that may affect embryogenesis, the cause of these anomalies is usually difficult to define. On the other hand, in laboratory, selected teratogenic factors are used to induce morphological changes in animals. Buczek (2000), Itow (1982), Itow and Sekiguchi (1980) obtained a variety of body deformities in arachnids in teratogenic experiments. Temperature is known to be a strong teratogenic agent. Embryo incubation at temperatures distinctly different from those which are preferred by a given species may disturb their development and lead to a range of anomalies: oligomely, schistomely, heterosymely, symely, bicephaly, and the combination of the above (complex anomalies) (Juberthie 1962; Jacuński et al. 2002a, b; Napiórkowska and Templin 2013a).

Generally, teratogenic studies focus on the morphology of individuals with developmental deformities. However, many of these anomalies are connected with anatomical changes, e.g., in the CNS (Harzsch et al. 2000; Jacuński et al. 2002a, b; Jacuński et al. 2005; Napiórkowska et al. 2010a; Scholtz et al. 2014). Studies which provide analysis of these changes are extremely important because they facilitate the classification of the obtained deformity. It must be remembered that morphological changes are not always reflected in the structure of internal systems, as has already been demonstrated by Jacuński et al. (2002a), Napiórkowska et al. (2013b, 2015), who studied *Eratigena atrica* (previous name: *Tegenaria atrica)*. In order to evaluate changes in the CNS of teratogenically affected *Eratigena atrica,* we therefore included histological analysis in the present study. We hypothesized that morphological changes would be consistent with changes in the structure of the ganglia. The objective of this study was to show the relationship between the morphology of the prosoma and the structure of the CNS.

## Materials and methods

Our teratological study involved embryos of *Eratigena atrica* (Agelenidae) from the breeding season 2016/2017, obtained from our laboratory culture. Sexually mature males and females, collected in the vicinity of Toruń and Chełmża (Poland), were transported to laboratory where they were kept in dark, at a temperature of 21–23 °C and humidity of about 70%. Each spider was kept in a separate glass container. Three weeks after mating, females laid cocoons, which were immediately removed from the containers and cut open to take out the eggs. The embryos z from each cocoon were counted and randomly divided into two groups. The control group was incubated in the conditions optimum for the embryonic development of this spider species, i.e., at the temperature of 23 °C and relative humidity of 70%. The experimental group was exposed to a teratogenic agent, i.e., alternating temperatures of 14 and 32 °C (changed every 12 h) applied for the first 10 days of embryonic development. The embryos were then incubated under constant thermal conditions, the same as those for the control group. Newly hatched spiders were examined for changes in the prosoma and opisthosoma; individuals with morphological deformities were photographed. Selected spiders were subjected to histological analysis, in which paraffin sections were stained with Meyer’s hematoxylin and eosin.

## Results and discussion

Approximately 2000 embryos were obtained in the breeding season 2016/2017. In the control group, where embryonic mortality was about 6%, no developmental anomalies were observed. In the experimental group, the mortality of embryos was high (about 30%) and a range of morphological anomalies were observed on the prosoma of the larvae. Seventy-eight out of 700 newly hatched larvae (approx. 11%) were affected by different deformities: oligomely, heterosymely, schistomely, polymely, and complex anomalies (Table 1).

**Table 1:** Kinds and frequency of anomalies in the prosoma in *Eratigena atrica*

Kind of anomaly	Number of individuals	%
Oligomely	45	57.69
Heterosymely	4	5.13
Schistomely	3	3.85
Polymely	1	1.28
Bicephaly	2	2.56
Complex anomalies	23	29.49
Total	78	100.00

**Fig. 1:**
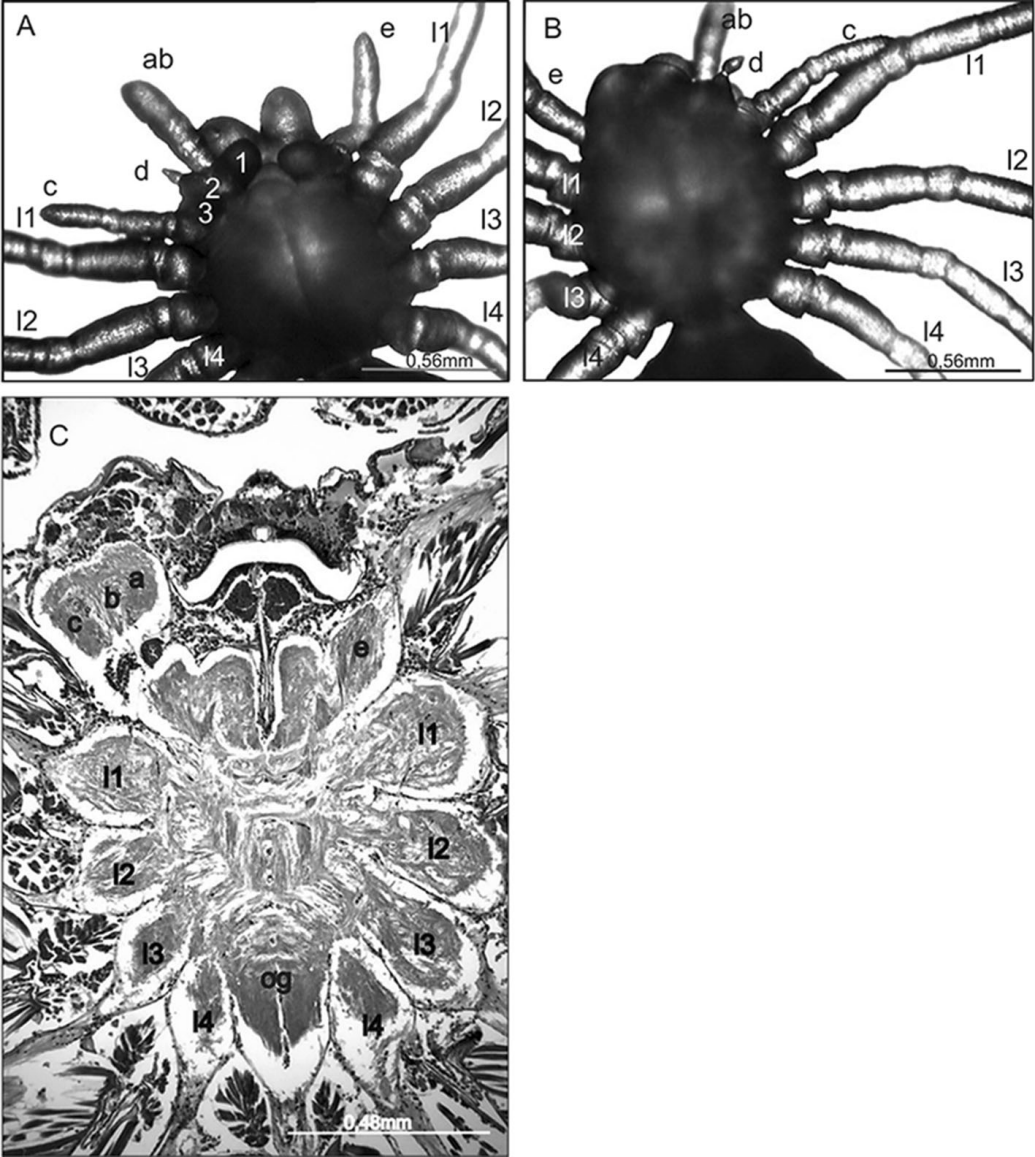
*Eratigena atrica* larva with complex anomaly. **a** Ventral view; **b** dorsal view: ab fused pedipalps, c properly built pedipalp, d stump, e pedipalp on unaffected side, l1–l4 walking legs, 1, 2, 3 gnathocoxae; **c** horizontal section through the prosoma and subesophageal ganglion (left side changed, opposite side correct): a, b, c fused ganglia of pedipalps ab and c, e ganglion of pedipalp on unaffected side, l1–l4 ganglia of walking legs, *og* opisthosomal ganglia

 From a group of 23 larvae affected by complex anomalies, we selected 4 larvae whose deformities were the most unique (extremely rare or not previously identified) for histological analysis. We expected to find changes in their CNS. However, only one individual had abnormalities in the subesophageal ganglion. In this spider, the complex anomaly affected the anterior part of the prosoma (Fig. 1a). On the left side of the prosoma (the ventral side) were three pedipalps (polymely) (a, b, c), two of which (a, b) were fused along their entire length (total heterosymely). The thickness of this fused structure indicated that it consisted of two (not just one) pedipalps. In addition, there were two well-formed gnatocoxae (1, 2) at its base. The segmentation of these appendages was not distinct, yet it was possible to distinguish six parts, two of which (distal) were slightly widened. Behind this fused structure was one properly built pedipalp (c) with its own gnatocoxa (3). In addition, on the dorsal side (Fig. 1b) the larva had a short club-shaped stump (d) between the fused pedipalps a, b and the pedipalp c. The remaining appendages (l1–l4) were fully formed with distinct segmentation.

Histological analysis of the CNS indicates significant changes in the anterior part of the subesophageal ganglion. On the affected side, there was a large mass, which consisted of three fused pedipalp ganglia (a, b, c) (Fig. 1c). No other changes in the shape and distribution of the remaining ganglia were observed.

The studied complex anomaly was for the first time obtained in teratological experiments using thermal shocks, although deformities of pedipalps had been reported before. In teratological experiments, these appendages tend to be malformed, missing (oligomely), bifurcated (schistomely), fused with chelicerae or walking legs (heterosymely), or supernumerary (polymely) (Jacuński et al. 2002a, b, 2004; Napiórkowska et al. 2016). Most often, teratogenically changed individuals are affected by only one anomaly. However, as we demonstrate in this study, complex anomalies of pedipalps are also recorded. The investigated larva of *Eratigena atrica* was affected by a range of defects: polymely (two additional pedipalps), total heterosymely (fusion of pedipalps), and had a short club-shaped stump, which, as was confirmed by histological analysis, was not innervated. At the same time, all three pedipalps on the affected side had their nerve ganglia. In the analyzed case, the fusion of appendages was reflected in the CNS. However, it is surprising that the ganglion of the free pedipalp was fused with the ganglia of the other two pedipalps. A similar case of two additional, fused pedipalps (polymely and heterosymely) has already been described by Napiórkowska et al. (2015), but in that case the two additional pedipalps were only partly fused. This structure had two ends of the same length, each consisting of two segments: tibia and tarsus. There was no stump on the prosoma. The histological analysis brought different results. On the right side of the prosoma with three pedipalps (including two heterosymelic), there were three separate ganglia in the CNS.

The previous and present studies indicate that morphological anomalies may have different consequences in the CNS. Complete and partial heterosymely of appendages was reflected in the CNS in two ways: (1) in the majority of investigated heterosymelic individuals (52) nerve ganglia were not fused, (2) only in two cases, heterosymely of the pedipalps and walking legs was connected with the fusion of corresponding ganglia (Napiórkowska et al. 2013b). Similar phenomenon accompanied polymely of walking legs (Napiórkowska et al. 2015). Although all supernumerary legs were free, their ganglia were fused in the subesophageal ganglion.

Our observations of a spider affected by a complex developmental anomaly confirm the hypothesis of different morphological and anatomical consequences of teratological changes.
